# Features of electronic and lattice mechanisms of transboundary heat transfer in multilayer nanolaminate TiAlN/Ag coatings

**DOI:** 10.1038/s41598-017-17291-4

**Published:** 2017-12-06

**Authors:** A. I. Kovalev, D. L. Wainstein, V. O. Vakhrushev, R. Gago, F. Soldera, J. L. Endrino, G. S. Fox-Rabinovich, S. Veldhuis

**Affiliations:** 1 Surface Phenomena Researches Group, I.P. Bardin Central Research Institute for Ferrous Metallurgy, 23/9 bdg. 2 Radio str, Moscow, 105005 Russia; 2 Instituto de Ciencia de Materiales de Madrid, Consejo Superior de Investigaciones Científicas, Sor Juana Inés de la Cruz, 3, 28049 Madrid, Spain; 3 Department of Materials Science and Engineering, Saarland University, 66123 Saarbruecken, Germany; 4 Surface Engineering and Nanotechnology Institute, School of Aerospace, Transport and Manufacturing (SATM), Cranfield University, College Road, Cranfield, Bedfordshire, MK43 0AL UK; 5 Department of Mechanical Engineering, McMaster University, 1280 Main St. W, Hamilton, ON L8S 4L7 Canada

## Abstract

Plasmon resonance heterogeneities were identified and studied along Ag and TiAlN layers within a multilayer stack in nanolaminate TiAlN/Ag coatings. For this purpose, a high-resolution plasmon microscopy was used. The plasmons intensity, energy, and depth of interface plasmon-polariton penetration were studied by scanning reflected electron energy loss spectroscopy. The heat conductivity of such metal-insulator-metal (MIM) nanolaminate coatings was measured by laser reflectometry. Dependencies of thermal conductivity coefficient of coatings, MIM interfaces, and resistivity of Ag layers as a function of the Ag-TiAlN bilayer thickness were calculated on the basis of experimental data. The contribution of plasmon resonance confinement to the abnormal lower thermal conductivity in the MIM metamaterial with Ag layer thickness below 25 nm is discussed. In particular, the results highlight the relevant role of different heat transfer mechanisms between MI and IM interfaces: asymmetry of plasmon-polariton interactions on upper and lower boundaries of Ag layer and asymmetry of LA and TA phonons propagation through interfaces.

## Introduction

Multilayer coatings of metal-insulator-metal (MIM) stacks result in 2D or 3D heterophase thin film composites that can be described as planar metamaterials. The properties of such materials are radically distinctive from the constituent phases. Growing activities in the study and development of new MIM systems can be explained by their outstanding fundamental properties as well as practical perspectives for tuning electromagnetic radiation in nanovolumes^1^. Interatomic exchange interactions at the metal-dielectric interfaces are implemented in this nanostructured material. In this way, MIM nanocomposites can acquire specific electromagnetic properties^2–5^ such as negative refraction index, permittivity ε_r_ and permeability μ_c_. Phonons, plasmons, and plasmon-polariton excitations developing in such materials are sharply distinct from ordinary metals or insulators. Phase composition, size and spatial distribution of building blocks in MIM control their response to external actions. For example, external electromagnetic radiation directly interacts with lattice and electronic subsystems in small resonant structures responding with a fine-adjusting signal. It has been shown that plasmon metamaterials can be designed for almost any wavelength range of the electromagnetic spectrum, from very low frequencies to UV^6,7^. The confinement and concentration of plasmon and plasmon-polariton resonance along the cross-section of the MIM metamaterials make these films interesting for optical applications^8,9^. Recently, new advances in nanofabrication of 2D and 3D multilayer heterostructures have resulted in an emerging field of plasmonics using metallic nanostructures to manipulate light^10^. In this case, the surface plasmon-polariton resonance (SPP) in metallic nanolayers and near the metal-isolator interfaces enables to transform light frequency. This property can be used to enhance the performance of energy-harvesting devices^11,12^ or could be applied for specific optical applications^13^.

Such MIM nanostructures have also another interesting feature: abnormally low heat conductivity^14^. The processes of light transmission and heat transfer in solids have common physical mechanisms that include propagation of phonon and plasmon oscillations in nanolayers and plasmon-polariton interactions at interfaces. Hence, these laminated materials present a multifunctional character by combining thermal barrier and optical properties. According to^14^, heat conductivity anomalies in MIM coatings produced by physical vapor deposition methods (PVD) arise from the alteration of individual components (phases) properties due to size effects and assembling in multi-layer coatings.

The thin film composites of interested for MIM require a sophisticated set of alternating layers of different compounds with a thickness from a few up to tens of nanometers. In this way, the distribution of individual layers in the cross-section of the nanocomposite is modulated. Heat conductivity of such material is controlled by the nature of the individual layers as well as the transfer mechanisms through the interfaces. The latter issue can be described by a Kapitza thermal boundary resistance resulting from phonon-phonon interaction^15^ or electronic disturbances. The roles of electron-phonon coupling or phonon-phonon coupling at interfaces still remain disputable^16,17^. Currently, the phonon contribution to the heat transport in metals and dielectrics is well known^18^, including heterophase multilayer coatings^19^. The experimental investigation confirmed the fact that the thermal conductivity of these materials is significantly attenuated when the size of the structural element becomes less than the mean free path of a phonon (10–100 nm)^20,21^. So, the phonon heat transport is significantly limited in nanolaminate systems. Under these conditions, plasmon-polariton and plasmon mechanisms of heat transfer acquire great importance. However, direct experimental studies of plasmon mechanism of heat transfer are scarce. This is partially motivated by the process complexity and the limitations of existing experimental techniques to study this issue. Theoretical models of heat transfer trough interfaces described in^22–24^ are very useful but have several limitations in their application to nanomaterials because they are derived from properties of “bulk” materials. The present research overrides difficulties of these models using plasmon microscopy. In this context, the main consideration is given to size effects and influence of interfaces onto plasmonic contribution in heat transfer of MIM coating. For this purpose, the features of the propagation of collective electron oscillations in nanovolumes and at interfaces were investigated in multilayer TiAlN/Ag coatings. Silver (Ag) was selected for metal nanolayers due to its high heat conductivity, while TiAlN was chosen as a hard and wear resistant dielectric material. Considerable attention has been focused on Ag nanolayers surrounded by a dielectric exhibiting strong size-dependent optical resonance in the visible light range from 1.8 to 3.3 eV at energies below the dielectric band gap (~4 eV). Such coatings are expected to be used as anti-reflecting wear resistant ones in solar energy applications.

## Results

In this work, we have used TiAlN/Ag multilayer coatings produced by direct-current magnetron co-sputtering using individual TiAl and Ag targets. The resulting structural properties of the TiAlN layers are described in detail elsewhere^25^, and their architectures are listed in Table 1.

**Table 1:** parameters of coatings under investigation.

Sample	TiAlN single layer thickness, nm	Ag single layer thickness, nm	Number of TiAlN/Ag bilayers	Total thickness, nm
1 (6BL)	40	40	6	480
2 (3BL)	150	150	3	900
3 (33BL)	20	5	33	825
4 (16BL)	20	10	16	480
5 (6BL)	150	20	6	1020
6 (8 BL)	30	30	8	480
TiAlN ref.	900	0	1	900

**Figure 1:**
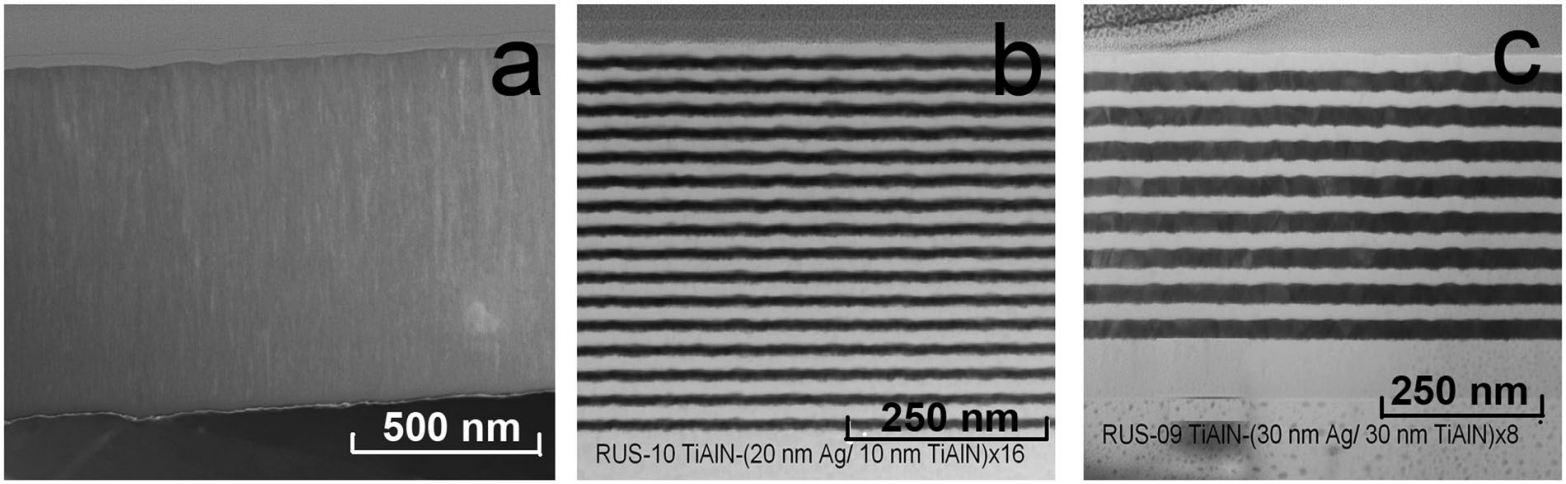
STEM image of TiAlN and TiAlN/Ag coatings with different composition prepared by DC magnetron sputtering: (**a**) TiAlN reference, (**b**) # 4, (**c**) # 6.

Figure 1(a–c) show STEM images of coatings #7, #4 and #6. The microphotographs evidence the homogeneous structure and uniform distribution of the individual layers. The Ag layers have a polycrystalline structure, while the TiAlN layers demonstrate (nano)columnar growth. Interfaces are well-defined, neglecting any layer intermixing.

It is well known that a heat transfer in solids could be realized by the electronic (plasmon) and lattice (phonons) mechanisms. The first one is typical for metals while the latter one occurs in dielectrics. As we have the metal-dielectric composites, we put our attention to studies of both mechanisms and their interaction.

**Figure 2:**
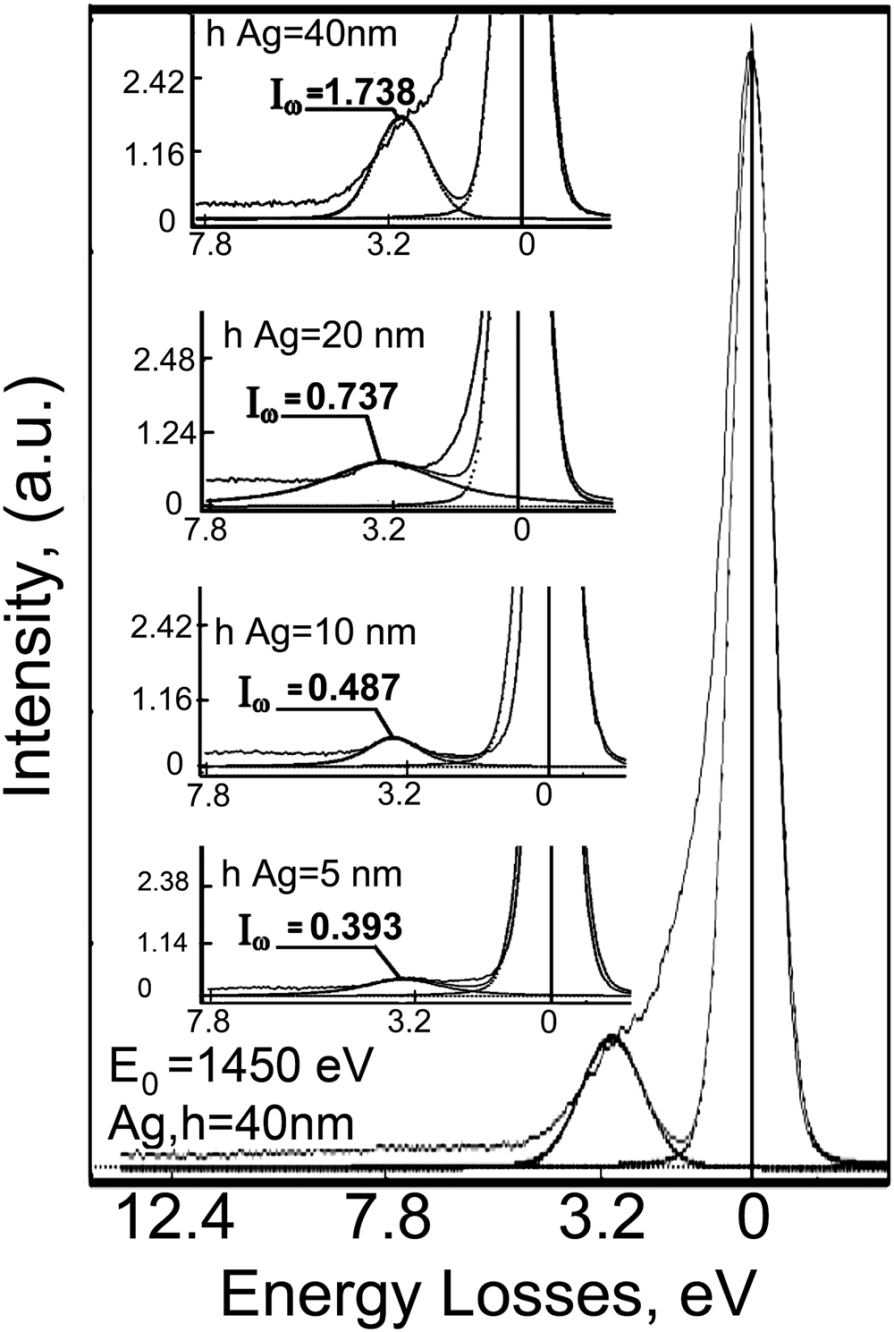
Plasmon peak intensities and excitation energies acquired from Ag layers with different thicknesses in TiAlN/Ag multilayer nanocomposite.

The peak of plasmon losses occurs near the line of back scattered electrons (E_0_ = 1450 eV) on distance 3.0–4.0 eV as shown in Fig. 2. The plasmon energy shifts to higher values whereas the plasmon intensity attenuates by decreasing the Ag layer thickness. According our data presented in^26^, the electronic structure of Ag layers is transformed in the nanometer range of thicknesses. The electronic states are localized and splitted, as was shown by the example of the localization of Ag 3d electrons and splitting of intraband transitions.

**Figure 3:**
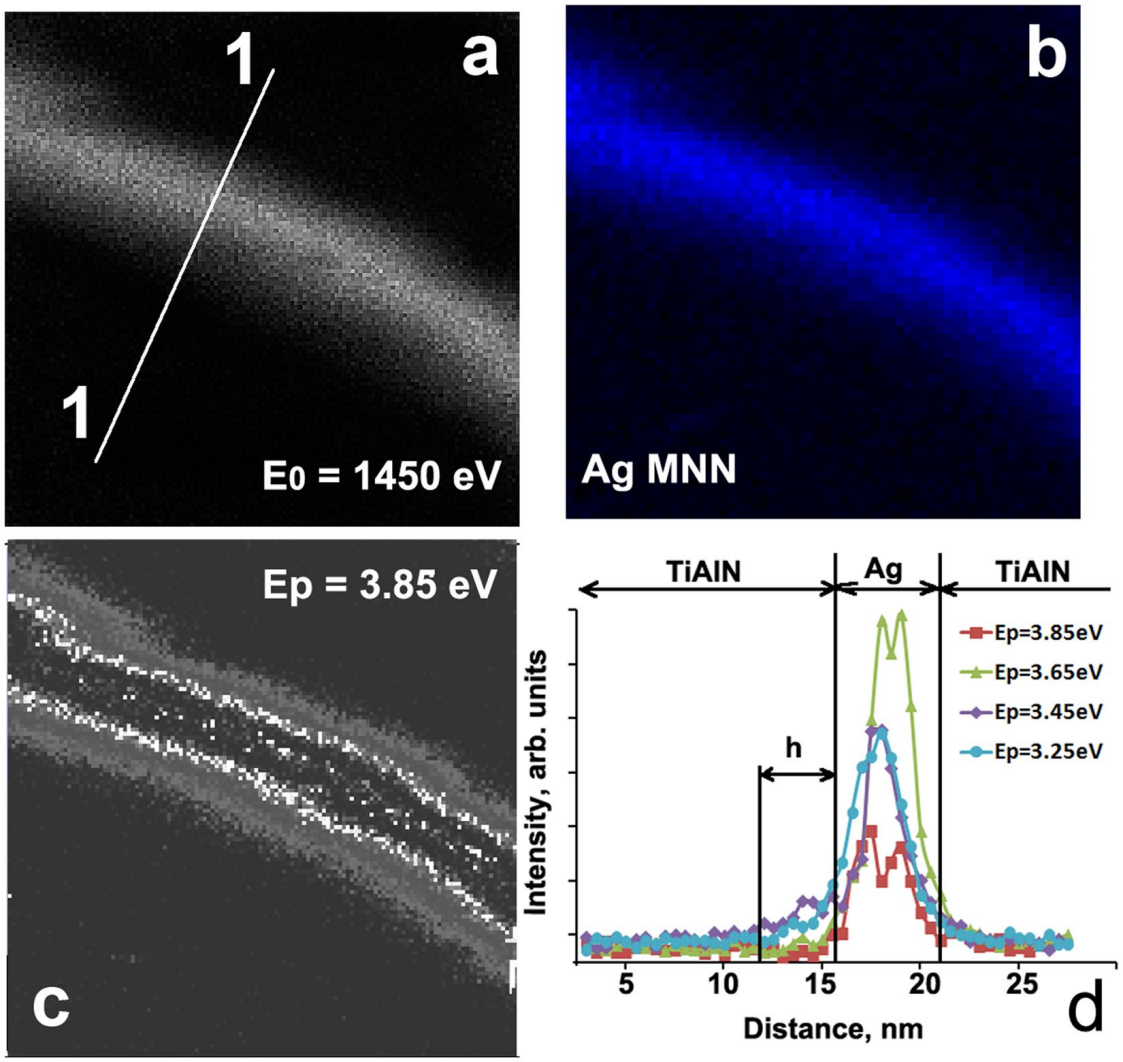
Scanning electron images (**a–c**) from a TiAlN/Ag (20/5 nm, #3) angle lap at fixed energies: (**a**) backscattered electrons, E_0_ = 1450 eV; (**b**) Ag MNN, E = 352 eV; (**c**) plasmon at ΔE = 3.85 eV with symmetrical plasmon resonance; (**d**) intensities of plasmon excitations along the line scan in a (1–1 direction) at different resonance energies.

Figure 3(a–c) are the illustrative electron microscopy images of an angled lap from the 20/5 nm TiAlN/Ag coating (#3) acquired with backscattered electrons (E_0_ = 1450 eV), AEM with Ag MNN Auger electrons (352 eV), and plasmon excitation at ΔE = 3.85 eV, respectively. Figure 3(d) shows the evolution of plasmon intensity at different energies from a line scan along the 1–1 direction on the angle lap (see Fig. 3(a)). As shown, the plasmon intensity distribution depends on the resonance energy. Plasmons are excited symmetric and antisymmetric in the silver layer depending on the energy of its excitation. These experimental observations correlate well with theoretical calculations fulfilled by J. Dionne *et al*.^27^. The terms “symmetric” and “antisymmetric” regarding plasmons intensities distribution in thin layers were used in this theoretical work.

Antisymmetric plasmon excitation at 3.25 and 3.45 eV propagates along phase boundaries. Such plasmons penetrate into the dielectric layer up to a certain depth *h* through the interface due to surface plasmon polariton (SPP) interactions. The picture of symmetric plasmon excitations with energy of 3.85 eV is shown in Fig. 3(c). This non-uniform resonance structure is a consequence of confinement of electromagnetic density of states in thin planar nanocomposite. Similar spatially inhomogeneous maps of plasmon excitations were observed earlier in the silver nanocrystals in a semi-empirical investigation^28^.

**Figure 4:**
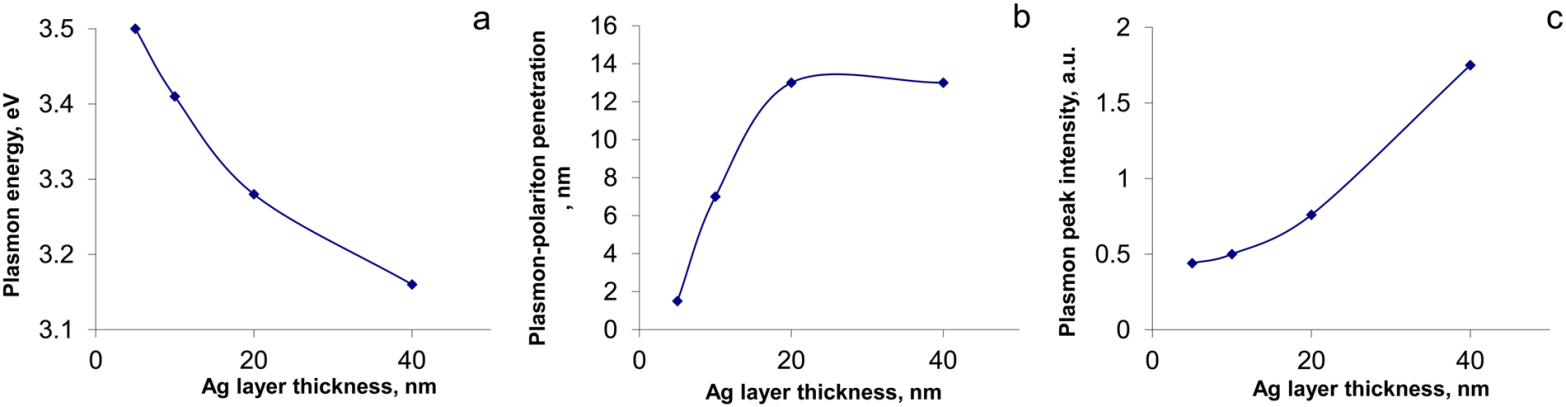
Influence of Ag layer thickness on the energy of plasmon REELS peak E_p_ (**a**), depth h of plasmon-polariton penetration at metal/dielectric boundary (**b**) and intensity I_p_ (**c**).

Based on the results from the relatively large number of TiAlN/Ag coatings with different architecture we can estimate the intensity (I_p_), energy (E_p_) of common plasmon pikes and maximal depth of interface plasmon penetration (h) in case of antisymmetric resonance (Fig. 4). Growth of plasmon resonance energy (Fig. 4(a)) with decreasing size of structure elements was observed earlier^29,30^. As one can see in Fig. 4(b), the depth of SPP penetration grows for lower plasmon energy as the Ag layer becomes thicker. This phenomenon is demonstrated by comparison of plasmons profiles with energies 3.85, 3.65, 3.45 and 3.25 eV in Fig. 3(d). Low energy plasmons at 3.45 and 3.25 eV have a lower possibility of inducing hot carriers and thermalization at nanometer distances. A similar situation can be found at other energy scales. For example, in thicker silver layers the energy of the plasmon peak decreases, its intensity goes up and, thus, the depth of plasmon-polariton penetration increases (see Fig. 2). The penetration depth of SPP into the TiAlN layer also depends on the intensity of the electromagnetic field generated by surface plasmons in the Ag layer. The resonance plasmon peak intensity grows as the Ag layer becomes thicker (see Fig. 4(c)).

SPP interactions could be the result of excitations from weakly bound electrons in dielectric or metal and its penetration into the dielectric.

**Figure 5:**
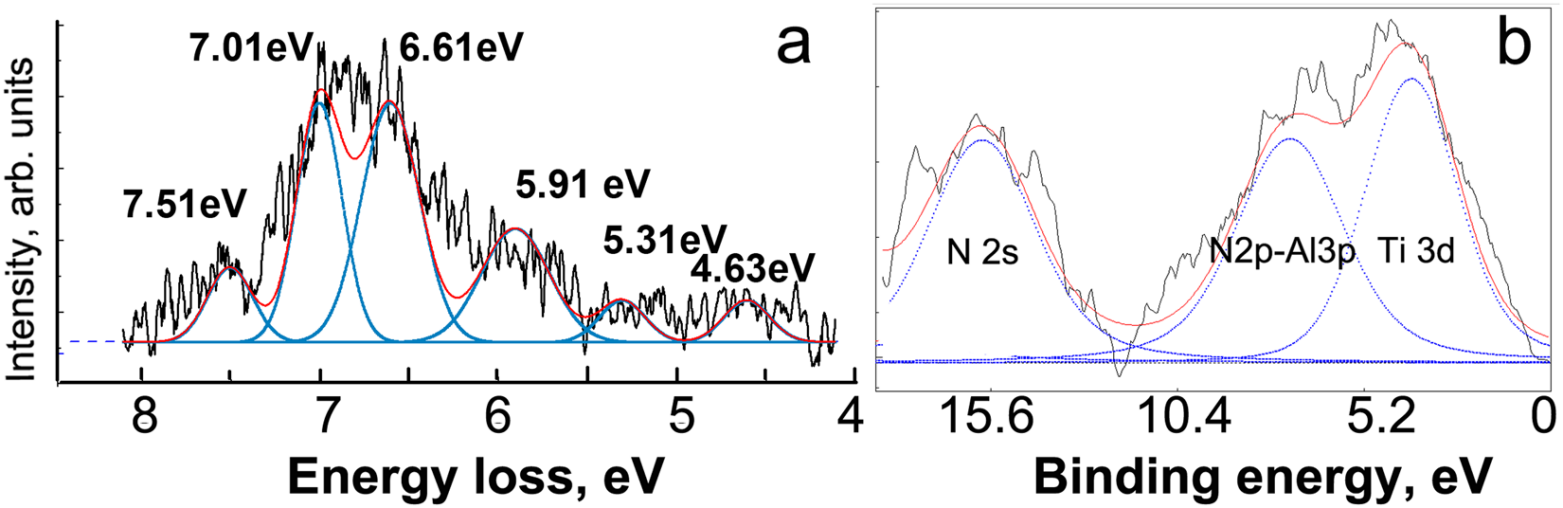
Interband transitions excited in TiAlN 20 nm layer obtained by HREELS (**a**) and XPS spectrum of TiAlN valence band (**b**).

Figure 5(a) shows HREEL spectrum of interband transitions in TiAlN 20 nm layer. From these data we can find out minimal energy of electron interband transitions between valence and conducting bands is 4.63 eV corresponding to TiAlN band-gap. The band gap in 100 nm TiAlN layers was in range of 3.9–5.1 eV^31^. XPS peak Ti 3d on Fig. 5(b) has binding energy 3.8 eV below the Fermi level. Splitting of Ti 3d and N 2p – Al 3d levels means that aluminum and nitrogen atoms are forming covalent bonds while Ti atoms arrange metallic bonds in the TiAlN. It is believed that these 3d electrons can participate in the SPP transboundary interaction.

**Figure 6:**
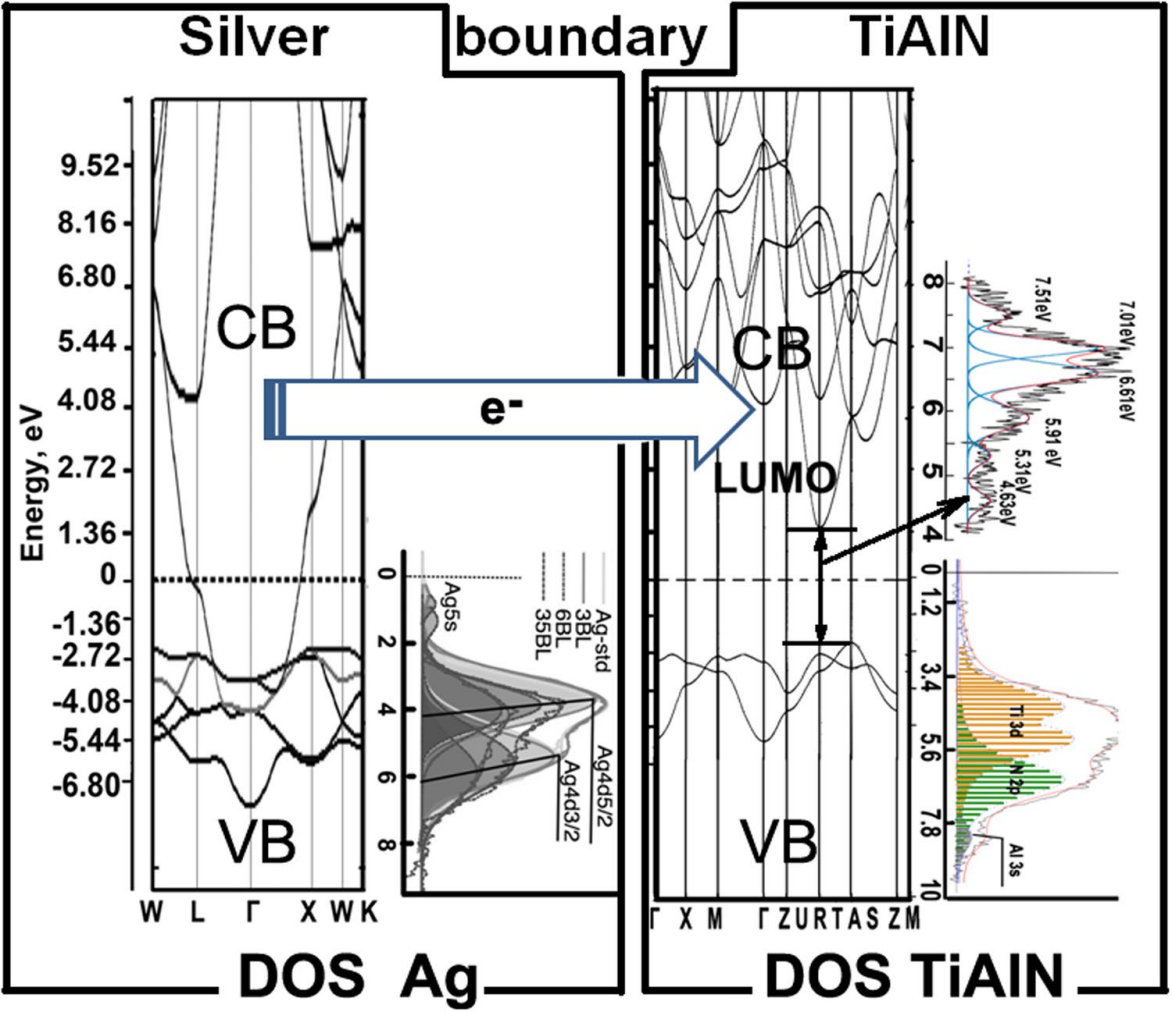
Scheme of SPP excitation on the Ag – TiAlN boundary. Band structures of TiAlN and Ag were taken from^31^ and^35^, correspondingly; Ag VB was citied from^39^; Interband transitions and VB of TiAlN were taken from our experiment (see Fig. 5(a,b)).

The localized electromagnetic field in the silver layer can excite collective oscillations of free electrons in the insulator. But this SPP excitation of Ti 3d electrons can appear at energy slightly higher than the band-gap energy. Another more probable way of SPP transboundary energy transition is a transport of Ag conducting electrons between metal and dielectric layers. Figure 6 shows a diagram of the proposed mechanisms of SPP excitation at boundaries in Ag/TiAlN multilayer coating as a combination of theoretical calculations and our own experimental results on fine structure of valence band (Fig. 5(b)) and interband transitions (Fig. 5(a)).

At the interface of the two layers under the influence of excitation by external energy source, metal conducting electrons can be injected into the conduction band of TiAlN. As it was been shown in previous paper^29^, Ag 5 s electrons are localized in silver nanolayers. This confinement reduces the electronic exchange interaction between the conduction bands of silver and nitride. This explains the decreasing of the intensity and depth of SPP penetration from Ag into TiAlN when the layer thickness decreases in the nanostructure region.

Plasmon-polariton transboundary interaction may explain the penetration of heat from the metal into the dielectric. Thermal barrier coatings of MIM properties will depend on the size of the pumping energy for the transition metallic conduction electrons in the insulator to overcome the energy barrier of the dielectric band gap. This barrier is reduced by the presence of Schottky barrier, which couples the metal and nitride. Its width can be about 1.0 eV; in particular it is 0.88, 0.92, 0.99 and 1.08 eV for Au, Pd, Ni and Pt^32^.

These electrons return back into the metal layer increasing the dissipation of energy at the interface.

Generally, intensity of plasmon oscillations in thin metal layers is lower than in thick ones. On this reason the effective depth of plasmon-polariton interaction is decreased with metal layer thinning. Lessening of intensity and depth of the plasmon-polariton interaction means a significant reduction in the concentration of conduction electrons and collapses the field of energy transmission from the metal to the dielectric at interfaces. In our case, the maximum depth of the plasmon - polariton penetration is reached at 12 nm. It was shown that the SPP depth into the dielectric medium δ_d_ for silver is approximately of 22 nm^33^. These calculations were carried out neglecting quantum effects in Ag layer. Hence, our results suggest that quantum phenomena in metal nanolayers can suppress the plasmon-polariton penetration at metal-dielectric interfaces.

The multilayer coatings can be represented as a repetition of n times a bilayer of metal and dielectric with thicknesses L_1_ and L_2_, heat resistivity of multilayer metamaterial R_ml_ and thermal conductivities K_1_ and K_2_, respectively. The layers are separated by a boundary with heat resistivity R_b_. So, the thermal conductivity of such MIM metamaterial (K_ML_) can be described as:

1 $${{\rm{K}}}_{{\rm{ML}}}=\frac{n({\rm{L}}1+{\rm{L}}2)}{{\rm{Rml}}}=(n(L1+L2))/((L1/K1+L2/K2)+(n-1){R}_{b})$$

Eq. (1) demonstrates that thermal conductivity decreases while the layer thickness comes smaller. Further, the total thermal conductivity K_ML_ of a solid is the sum of the phononic K_i_ and electronic K_e_ contributions:

2 $${{\rm{K}}}_{{\rm{ML}}}={{\rm{K}}}_{{\rm{e}}}+{{\rm{K}}}_{{\rm{i}}}$$

The calculated K_ML_ values for the relatively large number of coatings with different layer thicknesses and numbers of bilayers allow us to calculate thermal conductivity of interfaces and Ag nanolayer.

**Figure 7:**
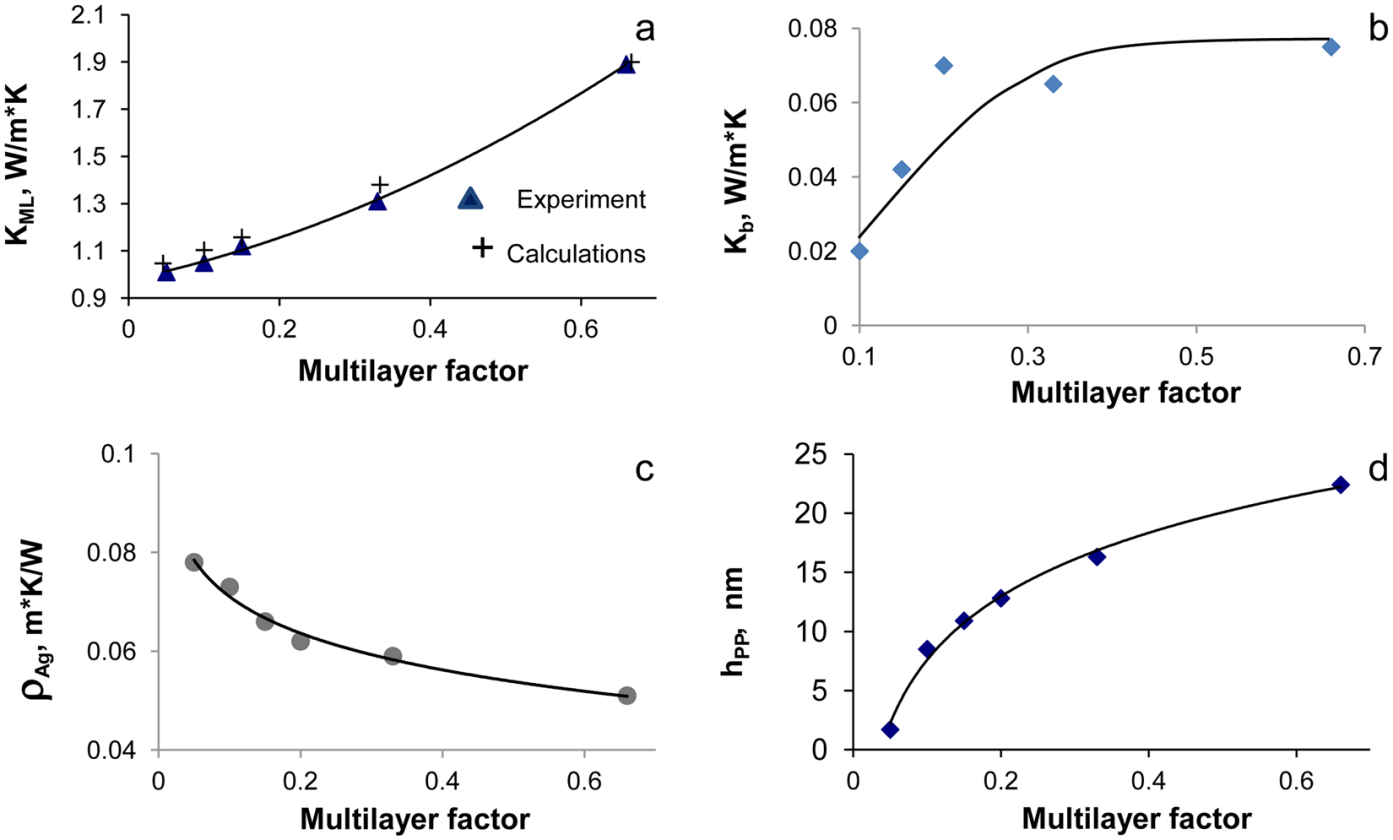
Dependence of thermal conductivities of multilayer coating K_ML_ (**a**) and Ag/TiAlN interfaces K_b_ (**b**); thermal resistivity of Ag layer ρ_Ag_ (**c**); plasmon-polariton penetration depth h (**d**) from multilayer factor X_ML_.

Figure 7 shows the experimental and calculated values based on Eq. (1) of the thermal conductivity vs. the multilayer factor that can be defined as:

3 $${X}_{ML}=\frac{({L}_{I}+{L}_{M})}{{N}_{BL}{L}_{M}}$$

The empirical parameter X_ML_ depends on the number of bilayers (N_BL_) in the MIM coating and the relation between total thicknesses of insulator (L_I_) and metal (L_M_). In this way, the multilayer factor X_ML_ decreases while the number of interfaces (N_BL_) grows.

One can see from Fig. 7(a) that the thermal conductivity of multilayer coatings (1–2 W/m·K) is nearly two orders of magnitude lower than that in bulk Ag (430 W/m·K) and below that from a thick monolithic TiAlN coating (2.96 W/m·K)^34^. According to Eq. (1), this may occur due to the size effects in TiAlN/Ag multilayer coatings where heat carried by the phonons (i.e., lattice thermal conductivity) in the dielectric and by plasmons in the metal (electronic part of thermal conductivity) is impeded at the interfaces by their thermal resistivity R_b_. The curves for K_b_ = 1/R_b_ and h are representing (Fig. 7(b,d)) the dependence of interface thermal conductivity and depth of plasmon-polariton penetration, respectively, as a function of the nanostructuring process. These dependencies are good correlated. The reduction of the depth of the plasmon-polariton penetration (h) is accompanied by a decreasing in the thermal conductivity of the metal-dielectric interfaces (K_b_). Shrinking of region near the metal-dielectric interface where the plasmon-polariton energy transfer is observed evidently decreases heat conducting probability. In other words, thermal transfer at interfaces depends on the intensity of the plasmon-polariton coupling. This interaction is realized by excitation of Ag conducting electrons propagated in TiAlN valence band (VB) in the electromagnetic field produced by plasmons in Ag layers. Inherently, this transfer of electronic excitation in the dielectric is analogous to a tunnelling effect, which plays an important role in the heat transfer of MIM nanocomposites.

However, the lessening in K_b_ (Fig. 7(b)) caused by interfaces is not sufficient to explain the observed experimental thermal conductivity K_ML_ shown in Fig. 7(a). It means that Ag thermal resistivity ρ_Ag_ of thin layers presented on Fig. 7(c) should grow with thinning of metallic layers inside the multilayer coating. Our calculations show that ρ_Ag_ plays an important role in the thermal conductivity of multilayer Ag/TiAlN coatings. As one can see on Figs 2 and 4, the plasmon intensity dramatically weakened during nanostructuring of silver layers. At the same time, the space resonance splitting is observed. Plasmon oscillations are excited in certain areas of the silver layer depending on their resonant frequency. Symmetric or antisymmetric type of plasmon resonance depends on Ag layer thickness. Theoretical studies have shown that the theoretical surface plasmon dispersion for a free-electron metal film surrounded by a dielectric is^35^:

4 $${\omega }_{s}^{\pm }={\omega }_{p}\sqrt{\frac{1\pm \exp (-q\cdot d)}{1+{\varepsilon }_{a}}},$$

where ω_s_ and ω_p_ are the frequencies of surface and volume plasmon, d is a thickness of the nanolayer and q is the wave vector. At small q · d only one branch can exist. Confinement of plasmonic resonance is a characteristic feature of silver nanolayers, and it explains partially the decrease in thermal conductivity occurring through electron-electron and electron-phonon dissipation of energy. The interfaces are an additional factor that significantly affects the heat transfer of multilayer nanocomposites. Plasmon-polariton interactions of the electronic subsystem are controlling the heat transfer between the metal and dielectric shrinking heat transfer cross-section.

The confinement is also a driving force of lattice thermal conductivity decreasing^36,37^. Dominant contributions of longitudinal acoustic (LA) phonons velocity to the thermal conductivity of planar nanostructures was presented in^19^. There we have reported about the degeneration of acoustic phonon oscillations with thinning of silver layers in Ag/TiAlN coatings below 150 nm. Earlier we have obtained phonon spectra in the set of Ag/TiAlN multilayer nanocoatings^26^ including ones from silver layers of various thicknesses. We continue analysis of these data in the present paper.

**Figure 8:**
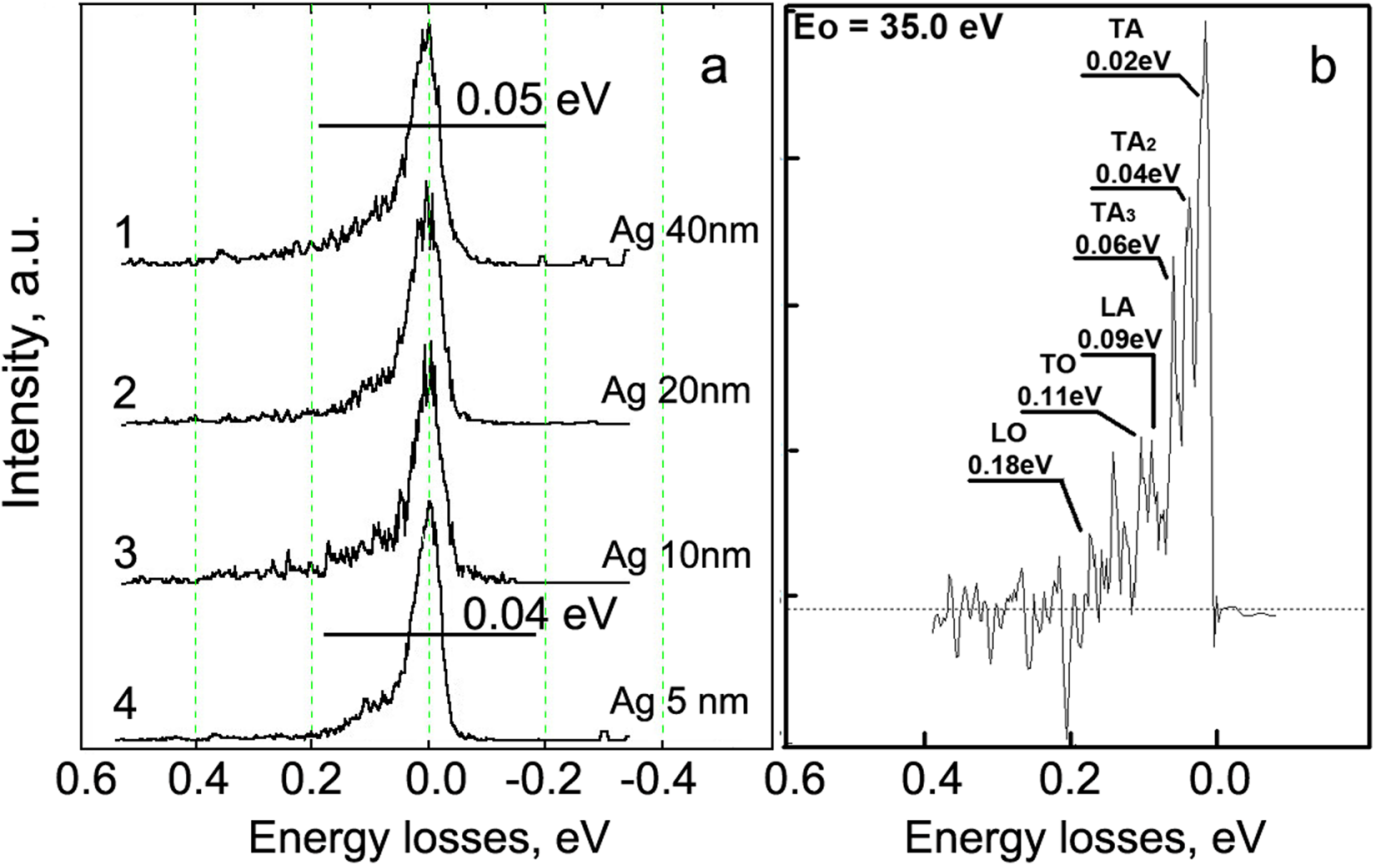
Phonon spectra of 5, 10, 20, 40 nm Ag layers (**a**) and differential phonon spectrum (**b**) calculated as (Spectrum 3 – Spectrum 4). E_0_ = 35 eV.

Figure 8 demonstrates the set of HREEL spectra of Ag layers (a) and differential phonon HREEL spectrum obtained by subtracting spectrum of Ag/TiAlN (5/20) from the spectrum of Ag/TiAlN (10/20) (b). One can see on Fig. 8(a) that half-widths (FWHM) of HREEL spectra are differ for each thickness of Ag layers. The level of noise on phonon spectra is enough low and can be estimated on spectra in the 0.3–0.4 eV energy losses range. The lessening of FWHM from 0.05 eV to 0.04 eV is a consequence of transverse acoustic (TA) waves attenuation clearly visible on Fig. 8(b). Evidently the “signal-noise” ratio of differential spectra is worse than on source ones but all features marked on Fig. 8(b) are physically meaningful. Thin layers are attenuating TA phonons while longitudinal acoustic (LA), transversal (TO) and longitudinal (LO) optical lattice vibrations are decreased to a lesser extent. However, the probability of phonon vibrations transmission through the metal-insulator interface has a greater attraction.

Thus significantly suppressed transverse phonon waves in comparison with longitudinal ones are the main cause of the reduction in the lattice thermal conductivity in MIM coatings with thin layers. Based on these results, we can calculate the probability of phonons propagation through an interface^23^:

5 $${a}_{1,j}(\omega )=\frac{{\nu }_{2,j}\,.\,{g}_{2,j}(\omega )}{{\nu }_{1,j}\,.\,{g}_{1,j}(\omega )+{\nu }_{2,j}\,.\,{g}_{2,j}(\omega )},$$

where α_ij_ is a phonon transmission probability of mode j in side 1, ω is a phonon angular frequency, v_1i_ is a phonon sound velocity of mode j in side 1, g_2j_ (ω) is a density of phonon state of mode j in side 2. This value corresponds to the intensity of the characteristic (i) phonon peak on the high-resolution spectra (see spectra in Fig. 8) and data published in^19^. The phonon propagation velocity was calculated as:

6 $${{\boldsymbol{\nu }}}_{i}={\omega }_{i}({\rm{GHz}}.{\rm{c}}^{-1})/{{\rm{q}}}_{{\rm{i}}},$$

where q_i_ is wave vector in Brillouin zone of TiAlN. This vector corresponds to q_i_ = $\frac{2\pi }{a\sqrt{3\,}}$ = 8.69 rad/nm for ГМ direction in reciprocal lattice. The wave vector q of Ag (ГL) $q=\frac{\sqrt{3}\pi }{a}$ = 13.3 rad/nm, where the parameters of silver crystal lattice a = 0.4085 nm. For simplicity, the thicknesses of silver and TiAlN layers were chosen equal in the calculations.

**Figure 9:**
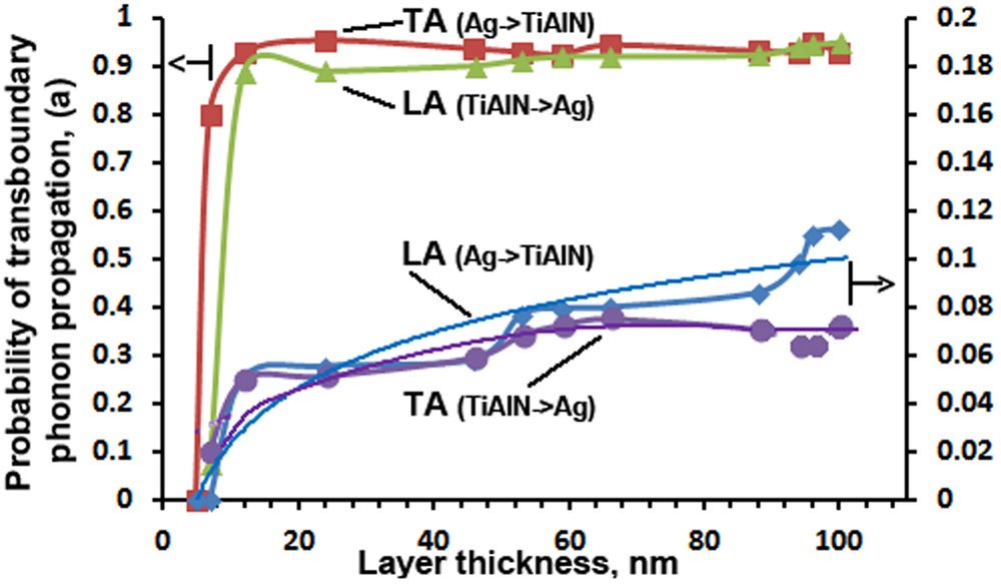
Phonon vibrations penetration probabilities through the interface between layers of the multilayer Ag/TiAlN coating on their thickness.

Figure 9 shows the calculated dependence of the probability of penetration of the phonon oscillations through the interface between metal (Ag) and insulator (TiAlN) layers as a function of the thickness of the layer. These calculations were made based on the analysis of experimental phonon resonance in Ag layers and TiAlN and Eq. (5). At 100.0 to 10.0 nm layers thickness, the probability of TA phonon propagation from silver into insulator is maximal, and it relies upon the thickness of the nanolayers minimally. The probability of LA lattice vibrations transfer from MI interface is increasing slowly with the layer thickness. The opposite pattern is observed for LA and TA phonon transboundary propagation in the IM interface. Such anisotropic penetration of acoustic phonons through the interfaces in MIM nanolaminate coating is stipulated by difference of elastic moduli of the layers, and it affects heat dissipation at the Ag-TiAlN interfaces and hampers heat propagation in a multilayer coating.

**Figure 10:**
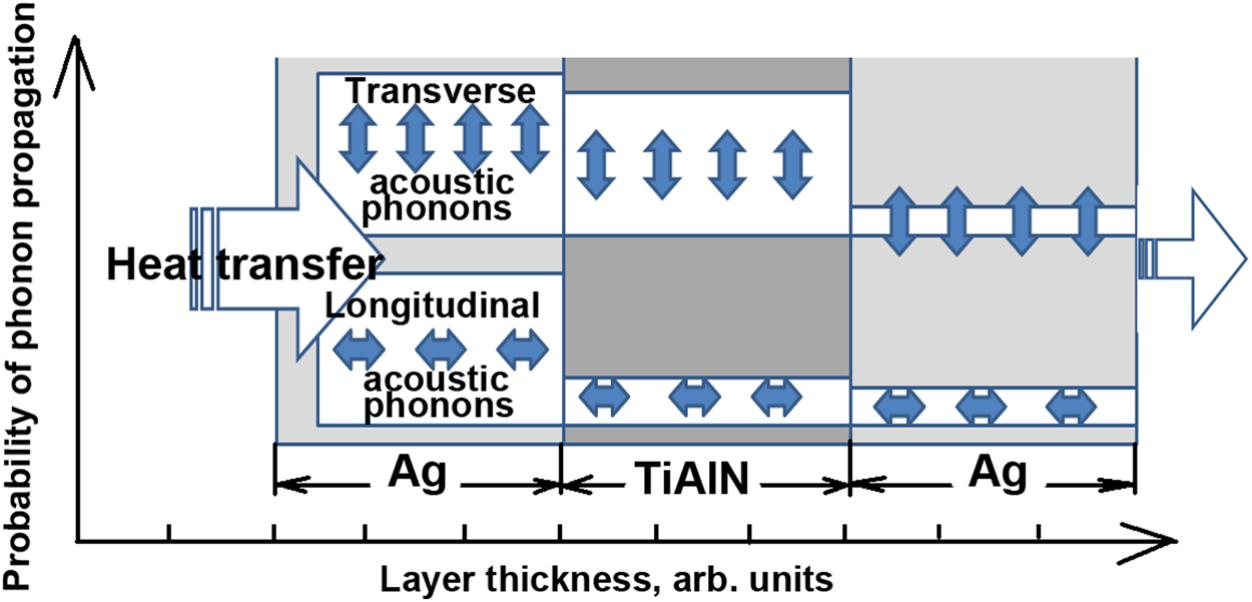
Scheme of phonon heat transfer in Ag/TiAlN nanolayer coating.

The scheme of transboundary heat transfer by phonons is drawn in Fig. 10. TA oscillations are negligibly attenuated when passing from Ag to the TiAlN layer, and they are damped by ten times during its transition from TiAlN to Ag. The observed asymmetry of the transboundary penetration of acoustic oscillations explains anomalous dropping of lattice thermal conductivity in MIM Ag/TiAlN planar heterostructure. This effect is reported for the first time and is essential for the understanding of the fundamental importance of physical processes of energy transfer through the interface into the metal-dielectric nanostructures.

## Discussion

As we have shown previously^14,29^, decreasing the nanolayers thickness radically affects the electronic structure of the metal, reduces the metallicity of interatomic bonds, and localizes valence electrons, thereby reducing the probability of their transport through the metal-insulator interface. This manifests itself in a noticeable reduction in the intensities of plasmon oscillations and the depth of penetration into a dielectric through the interface as it was shown by the experiment. Asymmetry of transboundary phonon heat transfer is an additional driving force weakening thermal conductivity in multilayer MIM coatings as it depends on a combination of many factors. Spatial splitting of the plasmon resonance, reducing the depth of the plasmon-polariton field at the interfaces, and quantum phenomena in nanolayers have been identified as the origin for the abnormal dropping of the electronic contribution to the thermal conductivity of the TiAlN/Ag coating. The change in the dielectric properties of the phases when incorporated in planar nanoscale heterostructures drastically affects the lattice heat transfer and, hence, results in a reduction for multilayer coatings.

Thermal conductivity of multilayer MIM nanomaterials is controlled by the combination of several factors as extracted from the study of TiAlN/Ag coatings. Spatial splitting of plasmons together with quantum phenomena in silver nanolayers suppress the depth of the plasmon-polariton excitations near the metal-dielectric interface and significantly affect the thermal conductivity of multilayer coatings. Distribution of the plasmon resonance intensities in the Ag nanolayers cross-sections and plasmon-polariton penetration depend on the Ag layer thickness, which was clearly demonstrated by plasmon electron microscopy.

Injection of free electrons from silver into TiAlN conduction band is the basis of transboundary SPP electronic mechanism of heat transfer. These results evidence some of the features of the heat transfer mechanism of MIM multilayers and open a new prospective for plasmon physics and engineering of plasmonic metamaterials. The asymmetry of LA and TA phonons propagation through interfaces in nanoheterostrucrures dramatically decreases the heat transfer in multilayer MIM coating, to be used as anti-reflecting wear resistant ones in solar energy applications.

## Methods

Scanning transmission electron microscopy (STEM) was used due to confirm the thickness and interface quality of the engineered nanolaminate coatings. The cross-sectional images were acquired from a lamella of the multilayer film prepared in a dual beam FEI Helios 600 by a focused ion beam (FIB) system using the “*in situ*” lift-out technique. The specimen was thinned to electron transparency, first, with an acceleration voltage of 30 kV and, later, with 5 kV (for at least 2 min. on each side) to minimize any possible preparation artefacts. The samples were then examined by STEM in the FEI Helios 600 system operating at 30 kV.

Thermal conductivity across the as-deposited coatings (from top to substrate) was measured by pulsed photothermal reflectance (PPR), as detailed in^38^.

The electron spectroscopy investigations were carried out with an electron spectrometer ESCALAB MK2 (VG) at vacuum 1·10^−8^ Pa. Scanning electron gun LEG200 was used in the measurements. Prior to the analysis, the surface of the sample was cleaned by Ar^+^ ions etching in the preparation chamber of the spectrometer at an argon pressure of 10^−4^ Pa, accelerating voltage of 8 kV, and current of 20 μA. Precise Ar ion etching was then used for depth profiling and sequentially expose the individual nanolayers and interfaces. Reflected electrons energy losses spectroscopy (REELS) was applied for the study of plasmons. Here, the electrons with a primary energy of 1450 eV are essentially inelastically scattered due to the excitation of vibrational and plasmon states of the first surface layers. This technique then records the spectrum of electrons scattered from the sample as a function of the energy loss, I(ΔE). The parameters of spectra acquisition were selected in the way to provide maximal energy resolution of the spectrometer. Auger electron microscopy (AEM) and electron microscopy of characteristic losses were used for imaging of elements and plasmons intensities distributions through the angle lap cross-section of multilayer coatings in individual layers and on interfaces at primary electron energy E_0_ = 1450 eV. Resolution of images was 256 × 256 pixels at × 10000 magnification. Topographic contrast was suppressed by image acquisition software.
